# Racial residential segregation and COVID-19 vaccine uptake: an analysis of Georgia USA county-level data

**DOI:** 10.1186/s12889-023-16235-0

**Published:** 2023-07-20

**Authors:** Simon K. Medcalfe, Catherine P. Slade

**Affiliations:** James M. Hull College of Business, Augusta University, Augusta, GA 30912 USA

**Keywords:** COVID 19, Vaccine uptake, Racial disparities in health, Racial residential segregation, Health policy

## Abstract

**Background:**

Foundational literature demonstrates that racial residential segregation results in poorer health outcomes for Black people than white people due to a variety of social determinants of health. COVID-19 vaccine uptake is important for better health outcomes, regardless of race. The COVID-19 pandemic has elevated concerns about racial health disparities but with little discussion of racial residential segregation as a predictor of disparate health outcomes. This paper investigates the relationship between racial residential segregation and COVID-19 vaccine uptake using county level data from the State of Georgia (USA).

**Methods:**

Using publicly available data, regression analysis is conducted for 138 of the 159 counties in Georgia USA, using a dissimilarity index that describes county level differences in racial residential segregation. The primary independent variable is Black-white differences in vaccine uptake at the county level. The analytic methods focus on a spatial analysis to support information for county level health departments as the basis for health policy and resource allocation.

**Results:**

Constructing a variable of the difference in vaccination rates between Black and white residents we find that Black-white differences in COVID-19 vaccination are most notable in the 69 most segregated of the 159 counties in Georgia. A ten-point lower segregation index is associated with an improvement in the Black-white vaccination gap of 1.5 percentage points (95% CI -0.31, -0.00). Income inequality and access to health care resources, such as access to a primary care physician, also predict Black-white differences in vaccination rates at the county level. Suggested mapping approaches of publicly available data at a state county level, provides a resource for local policy makers to address future challenges for epidemic and pandemic situations.

**Conclusion:**

County level and geospatial data analysis can inform policy makers addressing the impact of racial residential segregation on local health outcomes, even for pandemic and epidemic issues.

## Background

The seminal literature on the relationship between racial residential segregation and documented poorer health outcomes for Black persons has explored segregation as a modifiable risk factor in reducing racial health disparities [1]. Studies of this type have increased interest in longstanding concerns about the causes of racial health disparities [2] and Black persons’ health disadvantage in a neighborhood context [3]. Spatially focused research allows policy makers to address the community and social context aspects of the social determinants of health (SDOH) for better health for all.

COVID-19 has accelerated interest in awareness and understanding of Black-white differences in health outcomes including COVID-19 case and death rates [4–9]. Prior studies show that segregation is positively associated with case and death rates [4] but little research has examined the relationship between segregation or racial residential dissimilarity and COVID-19 vaccination rates.

It is widely accepted that vaccine development and uptake represent the difference between historical approaches to viral pandemics and the potential in the US to address COVID-19 and mitigate its long-term impact on individual and public health [10]. Several vaccines were developed for COVID-19, including those from pharmaceutical companies such as Pfizer, Moderna, and Johnson & Johnson. The vaccines became available to restricted groups in the US in December 2020 and to the majority of the population (those over 18) about March/April 2021. Although widely available from the spring of 2021, vaccine hesitancy affected the vaccine uptake results in geopolitical areas. Early research into vaccine uptake was primarily conducted via surveys of individuals before vaccines were available and the research explored whether people would or would not take the vaccine [11].

The SDOH variables found to be associated with vaccine uptake include education, housing insecurity, income, and urban/rural residency. Demographic factors associated with vaccine uptake include gender, age, and race/ethnicity [12–17]. Previous studies have also identified risk of exposure and severity of the disease as a contributing factor to vaccine uptake but with mixed results [18–23]. Current research still relies mostly on surveys and sampling at the individual level but there is a growing body of literature about spatial considerations of vaccine uptake at the county level [4, 24, 25] to support public health system response strategies [26]. Research that is more recent explores the relationship between vaccine hesitancy and vaccine uptake measured at the geopolitical level [4].

US resident attitudes toward COVID-19 vaccination and other related health behaviors are shaped by a complex combination of variables that predict both individual and shared population health [26]. Although there is some debate over the exact percentages, there is agreement that health outcomes are determined by social, economic, and environmental factors, health care, and individual health behaviors [27, 28]. Social, economic, and environmental factors are often grouped together and called SDOH. All agree that health care plays a minor role in health outcomes while Artiga and Heiman [15] and Schroeder [28] argue that the single greatest determinant of health outcomes is individual health behavior such as smoking and alcohol use. Fazilli [27] argues for SDOH. The SDOH framework is informative as it is the primary framework describing conditions in the environments where people are born, live, learn, work, play, worship, and age that affect a wide range of health, functioning, and quality-of-life outcomes and risks [29]. For example, prior literature addresses: 1) health outcomes such as morbidity [30]; 2) health behaviors such as childhood obesity [31]; and 3) in Georgia, the US state that is the target for this research, self-reported fair or poor health [32]. This framework is highly relevant to the impact of COVID-19 and the public health policy environment discourse [27, 28, 33].

Given the previous research identifying segregation as a social determinant of health, including COVID-19 outcomes, this study uses a novel approach to analyzing Black-white differences in COVID-19 vaccine uptake based on a meaningful county-level classification (most segregated versus less segregated) [1, 2]. Other county level covariates, including economic stability, education, healthcare access, neighborhood and physical environment, and health behaviors, are used to build a regression model that explores other social determinants of health that are associated with Black-white differences in vaccine uptake.

Public health officials and policy makers are concerned with both contagious disease impact on population health and the insidious health disparities that have continued for decades. The focus continues to be on systemic or structural racism with particular interest in racial residential segregation. Systemic or structural racism is defined as the sum of fundamental causes of health disparities that result from public policies and institutional practices that promote or reinforce racial inequality [2, 12]. A target for change continues to be racial residential segregation as it has been identified as a social determinant of pernicious health disadvantages for racial and ethnic minorities resulting from discriminatory policies at the local and regional geopolitical level. Racial residential segregation is a fundamental cause of health disparities that can be addressed by policy makers committed to improving the political power and access to resources for Black persons. This is especially true with respect to serious negative COVID-19 consequences for Black persons where systemic racism raises awareness of policies of disparate access to healthcare resources that resulted in racial inequities in COVID-19 infections and deaths [13].

The continuing discussion of systemic and structural racism suggests that we need a better-informed public health system, with core functions that include assessment, policy development, and assurance [5, 34]. This includes resource allocation [35] and providing targeted and focused life-saving information [36]. Public health governance has a mostly local structure and local public health officials are charged with addressing local needs using credible research such as COVID-19 vaccine uptake rates at the county level [5]. Health policy makers addressing allocation of resources and targeted communication strategies in their jurisdiction can use the basic analytic approach outlined in this paper using publicly available data [6, 7]. It can be descriptive of the lessons to be learned about systemic racism and health disparities from the COVID pandemic as well as prescriptive of the changes needed to address systemic racism that continues to result in racial and ethnic health disparities [14].

## Methods

### Data sources

Data on vaccination rates comes from the Georgia Department of Public Health (GDPH) [37] and represent the percentage of the county population documented as fully vaccinated. Note that our data was extracted on August 17, 2021, a week before Pfizer’s vaccine was fully approved by the FDA on August 23^rd^, 2021 and that vaccine uptake rates have been associated with FDA approval announcements [38]. Data on the social determinants of health, including community and social context, economic stability, education, healthcare access, neighborhood and physical environment, as well as health behaviors and county demographics was obtained from the University of Wisconsin Robert Wood Johnson County Health Rankings data for 2020 [39].

### Measures

County level community and social context variables include a Black-white segregation index and social association rate. Racial residential segregation is measured using an index of dissimilarity [40]. The index of dissimilarity measures how two groups (Black and white residents in this case) are distributed across census tracts that make up the county. The index ranges from 0 (complete integration) to 100 (complete segregation) and is interpreted as the percentage of Black or white residents that would have to move to a different census tract to produce a distribution that matches the county [39]. To see how the index of dissimilarity is constructed for a county, consider a county with two census tracks. The county population is 50% Black and 50% white. If all the Black residents live in one census track and all the white residents live in the other census track, then the county is completely segregated and has an index of dissimilarity is 100. If each census track is 50% Black residents and 50% white residents, then the index of dissimilarity is zero because each census track distribution of Black and white residents exactly matches the county. The social association rate measures the number of membership associations per 10,000 population. Membership associations include civic organizations, religious organizations and business and professional organizations [39].

Economic stability factors include unemployment, income, single parent homes, income inequality, and homeownership. Education variables include measures of high school and college education. Health care is represented by the primary care physician rate. Neighborhood and physical environment includes severe housing problems, access to exercise, and air quality [41, 42] Severe housing problems are defined as the percentage of households with at least 1 of 4 housing problems: overcrowding, high housing costs, lack of kitchen facilities, or lack of plumbing facilities. Access to exercise is the percentage of individuals who reside close to a location for physical activity such as a park or recreational facility. “Close” to a recreational facility is defined as one mile in an urban area or three miles in a rural area, or half a mile for a park. Air pollution was included in the regressions because COVID-19 is respiratory disease [41].

Health behaviors include obesity and flu vaccine rates. According to the Centers for Disease Control and Prevention (CDC) [43] adults with excess weight are at greater risk of severe illness and hospitalizations from COVID-19. Artiga and Hinton (2018) [15] state that individual behaviors such as diet and exercise have the largest impact on health and well-being, and that these behaviors are themselves influenced by social determinants of health. Lower income counties are less likely to have access to exercise facilities and healthy food which can contribute to poorer health behaviors such as obesity. Moreover, Bailey et al., (2017) [16] highlight how structural racism as a SDOH can impact health behaviors. This suggests that higher obesity rates in a county may not be the result of individual choice but systemic racism manifesting itself in segregated counties. The flu vaccine uptake has been linked to COVID-19 vaccine behavior. [44] Demographics include the proportion of Black and elderly residents as well as the total population of a county. Black and elderly persons have been shown to be disproportionately represented among COVID-19 deaths [45]. The variables are chosen based on previous research on social determinants of health, health behaviors, and demographics to avoid multicollinearity as shown in Table 1.

**Table 1:** Correlation coefficients between explanatory variables

		***1***	***2***	***3***	***4***	***5***	***6***	***7***	***8***	***9***	***10***	***11***	***12***	***13***	***14***	***15***	***16***	***17***	***18***	***19***	***20***	***21***
1	Segregation index	1.0																				
2	Social association rate	0.09	1.0																			
3	Pct unemployed	-0.18	-0.02	1.0																		
4	Log median household income	0.18	-0.17	-0.67	1.0																	
5	Pct single parent households	-0.11	0.24	0.60	-0.72	1.0																
6	Income ratio	-0.03	0.40	0.29	-0.49	0.53	1.0															
7	Pct homeowners	-0.22	-0.05	-0.33	0.34	-0.40	-0.19	1.0														
8	Highschool grad rate	-0.06	0.16	-0.21	0.12	-0.18	0.11	0.39	1.0													
9	Pct some college	0.23	-0.04	-0.42	0.71	-0.40	-0.22	-0.11	0.02	1.0												
10	Primary care physician rate	0.27	0.27	-0.11	0.26	-0.01	-0.00	-0.35	-0.03	0.47	1.0											
11	Severe housing problems	0.11	0.18	0.29	-0.43	0.61	0.31	-0.61	-0.33	-0.07	0.14	1.0										
12	Air pollution	-0.02	-0.12	0.12	0.14	0.08	-0.11	-0.08	-0.18	0.19	0.13	0.11	1.0									
13	Access to exercise	0.29	0.01	-0.26	0.47	-0.30	-0.30	-0.25	-0.23	0.46	0.46	0.04	-0.02	1.0								
14	Log case rate	0.10	0.13	0.09	-0.24	0.08	0.20	-0.26	0.07	-0.17	0.18	0.19	-0.19	0.03	1.0							
15	Log hospitalization rate	-0.07	0.32	0.34	0.55	0.56	0.35	-0.04	0.07	-0.51	-0.03	0.31	-0.14	-0.27	0.46	1.0						
16	Log death rate	-0.14	0.31	0.47	-0.63	0.58	0.39	0.03	0.09	-0.57	-0.08	0.21	-0.04	-0.32	0.20	0.70	1.0					
17	Pct flu vaccinated	0.27	0.04	-0.45	0.56	-0.47	-0.23	0.20	0.08	-0.17	-0.06	-0.18	0.09	0.34	-0.02	-0.23	-0.34	1.0				
18	Adults with obesity	-0.15	0.02	0.12	-0.24	0.27	0.21	-0.06	0.07	-0.17	0.18	0.07	0.04	-0.22	-0.04	0.15	0.23	-0.19	1.0			
19	Pct Black	-0.11	0.08	0.58	-0.46	0.78	0.32	-0.44	-0.31	-0.12	0.11	0.57	0.30	-0.16	-0.12	0.31	0.36	-0.45	0.16	1.0		
20	Pct 65 and over	-0.12	0.32	0.18	-0.36	0.30	0.18	0.41	0.19	-0.47	-0.18	-0.01	-0.17	-0.36	-0.06	0.37	0.43	-0.07	0.01	0.04	1.0	
21	Log population	-0.42	-0.10	-0.38	0.61	-0.33	-0.31	-0.29	-0.20	0.60	0.43	0.08	0.19	0.61	-0.07	-0.37	-0.42	0.36	-0.20	-0.06	-0.65	1.0

We also include terms that represent the prevalence of the COVID-19 disease in each county, the case rate, hospitalization rate, and the death rate. These rates were extracted from the GDPH website on January 7^th^, 2021 and therefore represent the prevalence of the disease in each county in the previous year. Recent research [46] has suggested cumulative cases, rather than daily cases, are more likely to influence appropriate risk responses. These measures of disease prevalence are not highly correlated as shown in Table 1. Log transformations were applied to the heavily skewed variables.

For the dependent variable, the GDPH provides two vaccination percentages we use in this study, the percentage of the white county population that is fully vaccinated and the percentage of the Black population that is fully vaccinated. We calculated the Black-white difference in vaccination uptake percentage (bwpctvaxdiff) for each county by subtracting the white vaccination uptake percentage from the Black uptake vaccination percentage.

### Statistical analysis

All analysis was conducted in Stata version 15 and the data is analyzed using ordinary least squares (OLS) regression. The estimated equation is as follows:

$$\bf bwpctvaxdiff\;=\;\alpha\;+\;\beta_1COMMUNITY\;AND\;SOCIAL\;CONTEXT\;+\;\beta_2ECONOMIC\;STABILITY\;+\;\beta_3EDUCATION\;+\;\beta_4HEALTHCARE\;+\;\beta_5NEIGHBORHOOD\;AND\;PHYSICAL\;ENVIRONMENT\;+\;\beta_6HEALTH\;BEHAVIORS\;+\;\beta_7COVID-19\;PREVALENCE\;+\;\beta_8\;DEMOGRAPHICS\;+\;error\;term$$

where the capitalized terms represent vectors of explanatory variables relating to SDOH, health behaviors, COVID-19 prevalence, and demographics; βs represent the vectors of coefficients of interest.

## Results

### Summary statistics

At the time of data extraction [37], 42.8% of the white resident Georgia population was fully vaccinated but only 39.0% of the Black resident population was vaccinated. However, eighty-one (81) counties had a Black vaccination percentage that was greater than the white percentage while the other seventy-eight (78) counties had white vaccination percentages higher than the Black percentage. This suggests that county level characteristics are important in determining vaccination rates.

The descriptive statistics are presented in Table 2 for the 138 counties with complete data and are grouped by SDOH categories. The dependent variable, bwpctvaxdiff, has a mean slightly below zero reflecting the slightly higher vaccination rate for white residents. The standard deviation, and minimum and maximum data show that there is wide variation across counties in terms of their vaccination rates for white and Black residents.

**Table 2:** Descriptive Statistics (*N* = 138)

	**Variable**	**Definition**	**Mean**	**Std. Dev**	**Min**	**Max**
**Dependent Variable**	bwpctvaxdiff	Percentage of the Black county population that is fully vaccinated minus the percentage of the white county population that is fully vaccinated	-0.097	9.530	-70.4	22
**Community and Social Context**	Segregation index	Index of dissimilarity where higher values indicate greater residential segregation between Black and white county residents	30.94	13.73	1.24	73.20
	Social association rate	Associations per 10,000 population	8.98	3.47	0	17.59
**Economic Stability**	Pct unemployed	Percentage of population ages 16 + unemployed and looking for work	4.44	.92	2.96	7.69
	Log median household income	The income where half of households in a county earn more and half of households earn less. (in logarithmic form)	10.73	.25	10.25	11.57
	Pct single parent households	Percentage of children that live in single-parent households	40.77	12.40	13.29	80
	Income ratio	Ratio of household income at the 80th percentile to income at the 20th percentile	4.97	.94	3.50	11.97
	Pct homeowners	Percentage of occupied housing units that are owned	67.97	8.95	26	86.01
**Education**	Highschool grad rate	Graduation rate	87.84	5.35	72.09	98.86
	Pct some college	Percentage of adults age 25–44 with some post-secondary education	50.72	11.96	20.75	81.57
**Health Care**	Primary care physician rate	Primary Care Physicians per 100,000 population	46.47	28.14	0	139.37
**Neighborhood and Physical Environment**	Severe housing problems	Percentage of households with at least 1 of 4 housing problems: overcrowding, high housing costs, or lack of kitchen or plumbing facilities	15.97	3.26	9.44	25.82
	Air pollution	Average daily amount of fine particulate matter in micrograms per cubic meter	10.74	.62	8.7	12
	Access to exercise	Percentage of the population with access to places for physical activity	54.17	26.79	0	100
**COVID-19 Prevalence**	Log case rate	Number of cumulative COVID-19 cases through January 7, 2021) per 100,000 county population (in logarithmic form)	8.58	.30	7.76	9.92
	Log hospitalization rate	Number of cumulative hospitalizations due to COVID-19per 100,000county population (in logarithmic form)	6.08	.48	4.81	7.53
	Log death rate	Number of COVID-19 deaths per 100,000county population (in logarithmic form)	4.73	.63	2.23	6.35
**Health Behaviors**	Pct flu vaccinated	Percentage of annual Medicare enrollees having an annual flu vaccination	41.13	5.62	25	53
	Adults with obesity	Percentage of adults that report BMI > = 30	34.45	5.89	23.4	57.7
**Demographics**	Pct Black	Percentage of population that is non-Hispanic Black or African American	27.95	17.32	.63	70.84
	Pct 65 and over	Percentage of population ages 65 and older	17.52	4.55	4.83	34.53
	Log population	Resident population. (in logarithmic from)	8.79	1.25	6.06	12.67

We are interested in the effect of racial residential segregation on COVID-19 vaccination uptake rates. Table 3 presents the mean values for our variables for the 69 most segregated and the 69 least segregated counties. The most segregated counties have a mean segregation index of 42.2 compared to 21.18 for the least segregated. The most segregated counties vaccinated Black residents at a 1.4 percentage point lower rate than white residents while the least segregated vaccinated Black residents at a 1.3 percentage point higher rate than white residents. The summary statistics also show how the most segregated counties differ from the least segregated counties. The most segregated counties are richer, have a lower unemployment rate, fewer single parent households, a higher primary care physician rate and access to exercise, have fewer adults with obesity and higher flu vaccination rates, and fewer Black residents. The more segregated counties have a higher COVID-19 case rate but the hospitalization and death rates are not statistically different.

**Table 3:** Mean values for most and least segregated counties

	**Variable**	**Most segregated**	**Least segregated**
**Dependent Variable**	bwpctvaxdiff	-1.38	1.33**
**Community and Social Context**	Segregation index	42.20	21.18***
	Social association rate	9.46	8.78
**Economic Stability**	Pct unemployed	4.23	4.50*
	Log median household income	10.80	10.72*
	Pct single parent households	38.39	43.21**
	Income ratio	4.90	4.99
	Pct homeowners	66.40	68.38
**Education**	Highschool grad rate	87.58	87.78
	Pct some college	53.00	49.90
**Health Care**	Primary Care physician rate	50.88	41.99*
**Neighborhood and Physical Environment**	Severe housing problems	16.16	16.04
	Air pollution	10.78	10.85
	Access to exercise	60.30	51.22**
**COVID-19 Prevalence**	Log case rate	8.67	8.55**
	Log hospitalization rate	6.08	6.10
	Log death rate	4.70	4.81
**Health Behaviors**	Pct flu vaccinated	43.46	39.33***
	Adults with obesity	33.63	35.92**
**Demographics**	Pct Black	24.81	31.83**
	Pct 65 and over	16.69	16.97
	Log population	9.30	8.71***

Means are statistically significantly different: *** *p* < 0.01; ** *p* < 0.05; *p < 0.1

**Fig. 1:**
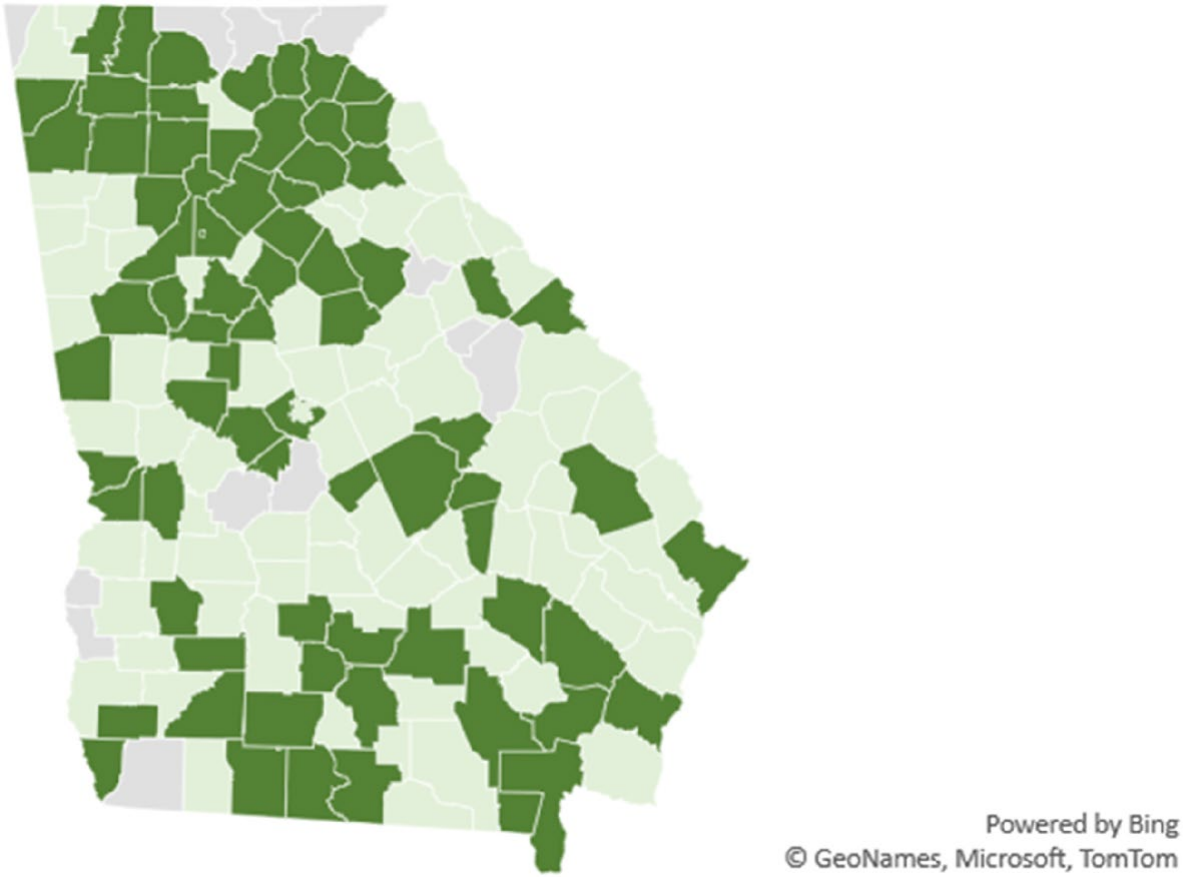
Most segregated (dark green) and least segregated counties (light green) in Georgia. No color represents missing data

**Fig. 2:**
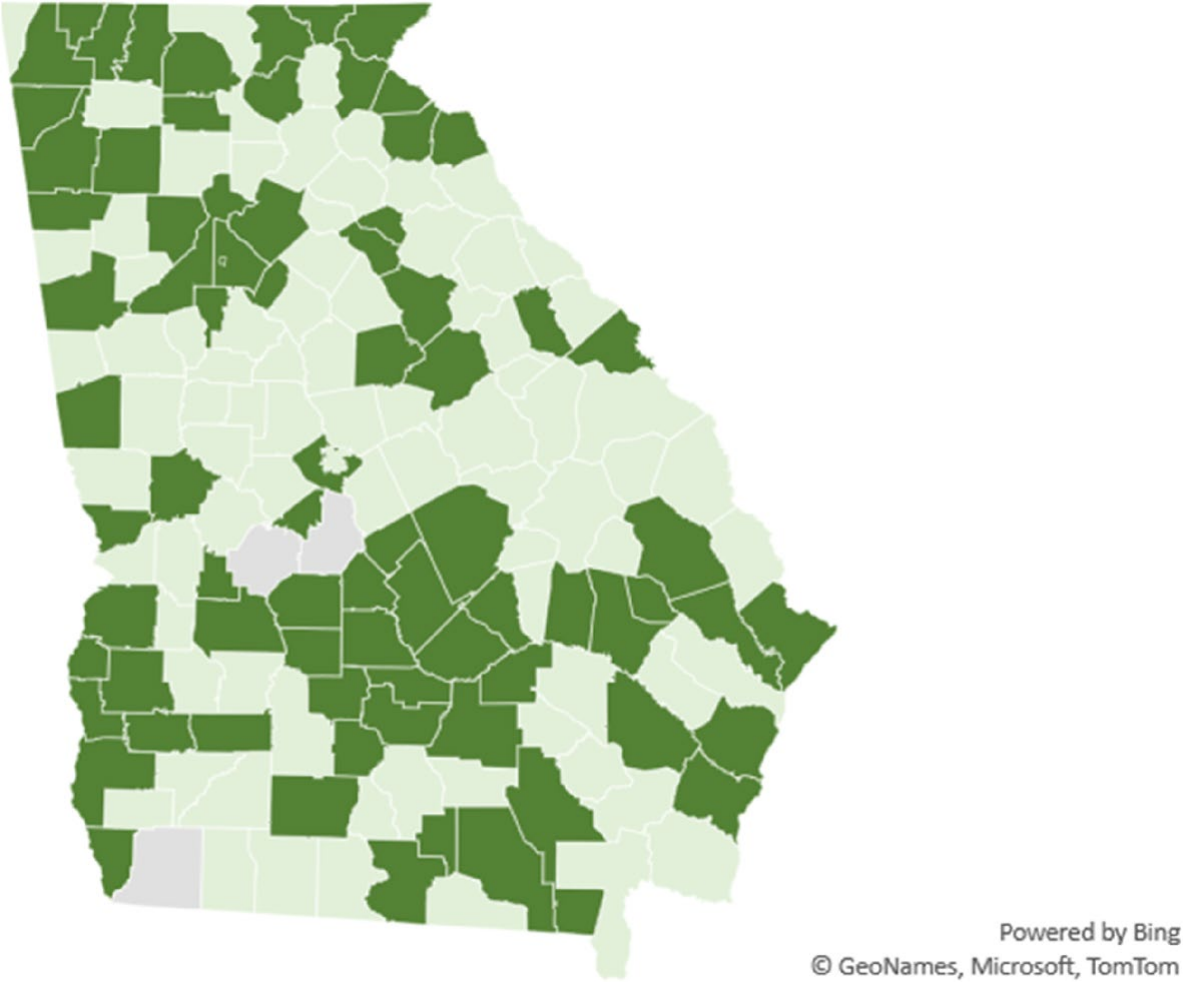
Counties that vaccinated Black (white) residents at a higher percentage than white (Black) residents in light green (dark green). No color represents missing data

Figure 1 is a map of the most and least segregated counties in Georgia and Fig. 2 is a map showing the counties that vaccinated Black residents at higher percentages than white residents. Visual inspection suggests there is a relationship between the most segregated counties also vaccinating white residents at a higher rate than Black residents. The next section of the paper formally tests for this relationship while controlling for other SDOH, health behaviors, COVID-19 prevalence, and demographics.

### Regression results

The OLS regression results are presented in Table 4. A post estimation test fails to reject the normality of the residuals, but heteroscedasticity was detected so robust standard errors are used to calculate confidence intervals. Column three (*All counties*) presents the results for all counties with complete data (*138 counties*) while column four (*Most segregated counties*) presents the results for the most segregated counties and column five (*Least segregated counties*) presents the results for the least segregated counties.

**Table 4:** Regression results

	**Variable**	**All counties**	**Most segregated counties**	**Least segregated counties**
**Community and Social Context**	Segregation index	-0.050 (-0.14, 0.04)	-0.153* (-0.31, -0.00)	-0.071 (-0.31, 0.17)
	Social association rate	0.325* (-0.06, 0.71)	0.337 (-0.30, 0.97)	-0.143 (-0.81, 0.52)
**Economic Stability**	Pct unemployed	-1.227 (-3.18, 0.73)	-1.219 (-4.54, 2.10)	0.105 (-2.26, 2.47)
	Log median household income	-1.566 (-14.34, 11.20)	-13.758 (-32.73, 5.21)	19.034** (1.82, 36.25)
	Pct single parent households	0.009 (-0.19,0.21)	-0.173 (-0.51, 0.16)	0.074 (-0.21, 0.35)
	Income ratio	-1.161* (-2.38, 0.06)	-3.150** (-5.96, -0.34)	-0.365 (-2.02, 1.29)
	Pct homeowners	0.309*** (0.08, 0.54)	0.393* (-0.01, 0.80)	-0.030 (-0.32, 0.26)
**Education**	Highschool grad rate	-0.063 (-0.28, 0.16)	0.029 (-0.34, 0.40)	0.129 (-0.18, 0.44)
	Pct some college	0.131 (-0.04, 0.30)	0.116 (-0.15, 0.38)	0.107 (-0.10, 0.31)
**Health Care**	Primary care physician rate	-0.038 (-0.08, 0.01)	-0.014 (-0.07, 0.42)	-0.116*** (-0.19, -0.4)
**Neighborhood and Physical Environment**	Severe housing problems	0.201 (-0.24, 0.65)	0.050 (-0.83, 0.93)	0.300 (-0.23, 0.83)
	Air pollution	3.696*** (1.79, 5.60)	3.355** (0.69, 6.02)	5.328*** (1.97, 8.68)
	Access to exercise	-0.012 (-0.08, 0.05)	-0.074 (-0.17, 0.02)	0.072* (-0.01, 0.15)
**Health Behaviors**	Pct flu vaccinated	-0.325*** (-0.54, -0.11)	-0.325 (-0.72, 0.07)	-0.530*** (-0.85, -0.21)
	Adults with obesity	0.005 (-0.17, 0.18)	-0.092 (-0.45, 0.27)	-0.000 (0.18, -0.18)
**COVID-19** **Prevalence**	Log case rate	-0.296 (-4.87, 4.28)	-1.341 (-9.32, 6.63)	2.087 (-4.64, 8.81)
	Log hospitalization rate	0.233 (-3.97, 4.43)	1.627 (-4.99, 8.25)	1.174 (-4.62, 6.97)
	Log death rate	-1.667 (-4.66, 1.32)	-2.150 (-8.54, 4.24)	-3.164 (-7.37, 1.04)
**Demographics**	Pct Black	-0.067 (-0.19, 0.06)	0.094 (-0.11, 0.29)	-0.150** (-0.30, 0.00)
	Pct 65 and over	-0.702** (-1.26, -0.14)	-1.043*** (-1.80, -0.29)	0.021 (-0.71, 0.75)
	Log population	-2.839*** (-4.74, -0.93)	-1.312 (-4.21, 1.59)	-5.217*** (-8.02, -2.42)
	Intercept	24.96 (-102.8, 152.8)	167.44* (-23.6, 358.5)	-216.82** (-397.3, -36.3)
	R-squared	.51	.67	.59
	Observations	138	69	69

^***^ *p* < 0.01; ** *p* < 0.05; *p < 0.1. 95% Confidence interval reported in parentheses

For the most segregated counties the segregation index is negatively associated with the Black-white vaccination rate. In other words, a lower segregation index is associated with more equal vaccination rates between Black and white residents. Specifically, a ten-point lower segregation index is associated with an improvement in the Black-white vaccination uptake gap of 1.5 percentage points. This improvement is enough to equalize the Black-white vaccination uptake gap of -1.38 in the most segregated counties. The other community and social context variable, the social association rate, is positive and marginally significant at the 10 percent level for all counties.

Considering the economic stability category for the most segregated counties, and all counties, the income ratio is also negative and significant. The least segregated counties have a significant and positive coefficient on the median household income variable.

The least segregated counties also have a negative and significant coefficient on the primary care physician rate, indicating that more physicians per capita in the county is associated with a worse vaccination uptake gap. The flu vaccination rate is also negative and significant. For the most segregated counties, and all counties, the percentage of the population over 65 is negative and significant.

Education and the prevalence of COVID-19 disease are not significant in the analysis. Air pollution was always significant and associated with an improvement in the Black-white vaccination uptake gap.

### Robustness checks

The maps in Figs. 1 and 2 suggest clustering of counties so analysis of county level data may need additional autocorrelation analysis. Spatial autoregression recognizes that neighboring counties influence each other. The results of applying spatial autoregression to the data set are presented in Table 5 and do not substantially change the results and conclusions draw from OLS regression.

**Table 5:** Spatial autoregressive results

	**Variable**	**All counties**	**Most segregated counties**	**Least segregated counties**
**Community and Social Context**	Segregation index	-0.04	-0.13*	-0.15*
		(-0.12, 0.03)	(-0.28, 0.02)	(-0.31, 0.01)
	Social association rate	0.29* (-0.04, 0.62)	0.40 (-0.15, 0.95)	-0.34 (-0.79, 0.11)
**Economic Stability**	Pct unemployed	-0.99	-0.82	-0.54
		(-2.53, 0.54)	(-3.73, 2.09)	(-2.26, 1.19)
	Log median household income	-0.56 (-9.99, 8.87)	-13.91* (-29.89,2.07)	17.91*** (6.80, 29.02)
	Pct single parent households	0.01 (-0.16, 0.18)	-0.21 (-0.48, 0.06)	0.01 (-0.18, 0.20)
	Income ratio	-1.21**	-3.35***	-0.20
		(-2.38, -0.03)	(-5.46, -1.25)	(-1.43, 1.03)
	Pct homeowners	0.29***	0.36**	-0.12
		(0.08, 0.49)	(0.04, 0.68)	(-0.35, 0.11)
**Education**	Highschool grad rate	-0.04 (-0.25, 0.17)	0.04 (-0.27, 0.34)	0.15 (-0.09, 0.39)
	Pct some college	0.13*	0.12	0.03
		(-0.00, 0.25)	(-0.08, 0.32)	(-0.10, 0.17)
**Health Care**	Primary care physician rate	-0.04* (-0.09, 0.00)	-0.02 (-0.08, 0.05)	-0.10*** (-0.16, -0.04)
**Neighborhood and Physical Environment**	Severe housing problems	0.08 (-0.33, 0.49)	0.08 (-0.65, 0.82)	0.26 (-0.12, 0.64)
	Air pollution	2.76***	2.84**	2.92*
		(1.13, 4.39)	(0.66, 5.03)	(-0.03, 5.87)
	Access to exercise	-0.02	-0.07*	0.07**
		(-0.07, 0.03)	(-0.15, 0.00)	(0.02, 0.13)
**Health Behaviors**	Pct flu vaccinated	-0.32***	-0.33**	-0.50***
		(-0.51, -0.12)	(-0.63, -0.03)	(-0.73, -0.28)
	Adults with obesity	0.02	-0.08	-0.01
		(-0.13, 0.18)	(-0.34, 0.17)	(-0.16, 0.14)
**COVID-19 Prevalence**	Log case rate	0.35	-2.64	-3.03
		(-3.68, 4.38)	(-9.46, 4.19)	(-9.08, 3.02)
	Log hospitalization rate	-0.11 (-3.11, 2.88)	1.34 (-3.27, 5.96)	2.44 (-1.07, 5.95)
	Log death rate	-1.57	-2.42	0.07
		(-4.04, 0.90)	(-6.77, 1.93)	(-3.11, 3.26)
**Demographics**	Pct Black	-0.04	0.10	-0.14**
		(-0.14, 0.06)	(-0.06, 0.27)	(-0.26, -0.03)
	Pct 65 and over	-0.67***	-1.12***	0.06
		(-1.0, -0.26)	(-1.77, -0.47)	(-0.42, 0.53)
	Log population	-2.87***	-1.85	-3.69***
		(-4.36, -1.39)	(-4.47, 0.76)	(-5.86, -1.51)
	Intercept	17.57	194.18**	-157.46**
		(-79.9, 115.1)	(33.6, 354.8)	(-301.2, -13.7)
	R-squared	.50	.68	.49
	Observations	138	69	69

^***^ *p* < 0.01, ** *p* < 0.05, * *p* < 0.1. 95% Confidence interval reported in parentheses

The main difference from the OLS regressions is that segregation is negative and significant for both the most and least segregated counties.

We also consider backwards stepwise OLS regression (*p* > 0.2) and the results confirm the negative association between racial residential segregation and the Black-white vaccination uptake gap for the most segregated counties (see Table 6). Other independent variables coefficients and significance are consistent with the OLS and spatial autoregressive results presented in Tables 4 and 5.

**Table 6:** OLS stepwise regression results

	**Variable**	**All counties**	**Most segregated counties**	**Least segregated counties**
**Community and Social Context**	Segregation index		-0.16** (-0.30, -.02)	
	Social association rate	0.33* (-0.02, 0.69)		
**Economic Stability**	Pct unemployed	-1.55* (-3.25, 0.15)	-1.90 (-4.76, 0.96)	
	Log median household income		-10.94 (-26.93, 5.04)	20.32*** (11.45, 29.19)
	Pct single parent households			
	Income ratio	-1.19** (-2.15, -0.22)	-2.95** (-5.34, 0.55)	
	Pct homeowners	0.27*** (0.14, 0.41)	-0.37** (0.06, 0.68)	
**Education**	Highschool grad rate			
	Pct some college	0.10 (-0.03, 0.24)	0.18* (-0.01, 0.37)	
**Health Care**	Primary care physician rate	-0.05** (-0.08, -0.01)		-0.09*** (-0.15, -0.03)
**Neighborhood and Physical Environment**	Severe housing problems			0.34 (-0.08, 0.75)
	Air pollution	3.61*** (1.95, 5.26)	3.27*** (1.11, 5.43)	4.09*** (1.27, 6.90)
	Access to exercise		-0.08* (-.17, 0.01)	0.05* (-0.01, 0.11)
**Health Behaviors**	Pct flu vaccinated	-0.31*** (-0.51, -0.11)	-0.31** (-0.61, 0.02)	-0.43*** (-0.67, -0.20)
	Adults with obesity			
**COVID-19 Prevalence**	Log case rate			
	Log hospitalization rate			
	Log death rate	-1.60 (-3.96, 0.75)		-1.77 (-4.15, 0.59)
**Demographics**	Pct Black			-0.11*** (-0.18, -0.34)
	Pct 65 and over	-0.73*** (-1.22, -0.24)	-0.97*** (-1.48, -0.46)	
	Log population	-3.35*** (-4.67, 2.03)	-1.52 (-3.54, 0.48)	-4.91*** (-6.25, -3.56)
	Intercept	11.97 (-20.03, 43.96)	125.49 (-24.91, 275.89)	-193.16*** (-286.00, -100.32)
	R-squared	.49	.65	.56
	Observations	138	69	69

^***^ *p* < 0.01, ** *p* < 0.05, * *p* < 0.1. 95% Confidence interval reported in parentheses

Overall, the robustness checks confirm the main conclusion that racial residential segregation is associated with larger Black-white differences in COVID-19 vaccination rates in the most segregated counties in Georgia, USA.

## Discussion

Using county level data from the state of Georgia (USA) this study presents evidence that more segregated counties have larger COVID-19 vaccination rate differences between Black and white residents. Other factors associated with COVID-19 vaccination racial disparities include income, income inequality, health care access and health behavior, as well as social associations. These results support previous research that found segregation was an important determinant of COVID-19 case and death rates [4, 47]. It also supports the ongoing discussion of structural and systemic racism such as racial residential segregation that must be addressed with specific interventions by policy makers if pernicious racial health disparities are to be addressed in a fundamental way [48, 49]. County level analysis is useful to inform public health policy that is mostly determined at the county level [5, 34]. Using this analytic and mapping approach public health officials can target and direct resources to specific racial and ethnic populations in counties based on their current needs and racial disparity issues [50].

The findings on economic stability differences suggest that income inequality manifests itself in vaccination rate inequality. Further, given that median household income is lower in the least segregated counties, this suggests that they utilize their scares resources better to reduce the vaccination gap.

We suggest three strategies that should be considered by local public health officials based on the results of this study. First, social association in our results suggest a positive relationship with Black-white vaccination differences [51]. Therefore, communication through community organizations, including civic, religious, sports, political and professional work-related organizations may reduce health disparities at the county level [33, 36, 52].

Second, systemic racism focuses on disparate access to resources. Our findings are relevant since those counties with persons aged over 65 had access to the vaccines earlier than the rest of the population. This suggests that white older residents may have been vaccinated at higher rates than Blacks older residents. Moreover, the most segregated counties have more primary care physicians per capita but vaccinate Black residents at a lower percentage than white residents. Taken together, these results suggest that access to health care is an issue, it is not the number of physicians in a county that narrows the Black-white vaccination uptake gap, but access to those physicians. If physicians’ offices and vaccination centers are not located in areas of the county that can be accessed by all residents, because of systemic racism creating segregated counties, then some members of society may be disproportionately affected.

Third, the level and distribution of income are important co-variates with vaccination rates. The least segregated counties are better able to use lower income levels to allocate resources more equitable. On the other hand, the most segregated counties have higher income inequality associated with greater Black-white vaccination differences.

Additionally, some results, such as air pollution being associated with a smaller Black-white vaccine uptake gap require further research. If Black persons live in parts of the county that have greater air pollution, they may be more inclined to get the vaccination and therefore the Black-white vaccination uptake gap decreases. Alternatively, urban areas may have more Black residents and more pollution [41] but also more sites to get vaccinated. This is an opportunity to further explore rural versus urban differences in systemic racism including access to healthcare resources.

The strengths of our study are that we use county level data rather than individual data. This means our results have public policy implications that can be analyzed and implemented at the county level. Recent research [7] suggests clustering counties by similar characteristics allows for targeted responses by policy makers, which is the priority for addressing pernicious systemic racism. Our novel clustering is by segregation and more segregated counties vaccinate Black residents at lower rates than white residents. To address racial health disparities in particular, public policy and resources should be targeted toward these counties [53]. In the short run, this may result in a greater emphasis on reaching segregated communities with vaccinations. In the long-run, reducing segregation itself may have long term beneficial health effects for all health behaviors and outcomes [14].

This study is not without limitations. We use county level data from Georgia but it is not obvious that the same relationship between segregation and vaccination uptake rates would hold in other states in the United States or other countries. Although we find associations between Black-white differences in vaccination uptake rates and a variety of SDOH, including segregation, consistent with recent findings on socioeconomic disparities in COVID-19 vaccine uptake and in particular second and booster doses designed to improve immunity [47], correlation does not imply causation. It should be noted that we only consider Black versus white people differences and we recognize that there are many other people of varying races and ethnicities where research would be appropriate for policy development. We also recognize that our work here does not predict vaccine uptake at the individual level, including the reasons that an individual may not choose vaccination. That is not the intent of the study. Nevertheless, our results still encourage public health officials to use our approach and findings on Black-white differences to analyze readily available data at the geospatial level to allocate resources to encourage vaccination of their constituents and stakeholders.

## Conclusion

Racial health disparities have been an ongoing concern in the United States for many years. COVID-19 has similarly disproportionately affected people of color. Vaccinations are one health response to combating health issues specifically and health disparities in general. Unfortunately, our study finds that the most segregated counties in Georgia have larger Black-white differences in vaccination rates with Black residents in the disparate position. Considering the impact of racial residential segregation on disease prevention such as vaccine uptake, resources may be allocated by public health officials to certain more-segregated communities to combat future strains of Coronavirus as well as other population health challenges. Our research supports the growing call for better understanding of the underlying causes of health disparities, including those policies that promote racial residential segregation and its consequences for Black-white differences in health outcomes. Policies that promote fundamental causes of health disparities require more attention and a concerted research and policy-response effort. Despite decades of research on racial and ethnic disparities in a wide variety of health outcomes, pernicious health disparities exist. Using COVID-19 vaccine uptake, our study contributes to the growing conversation about the need for more attention, resources and progress to reduce racial and ethnic health disparities. Our research approaches racial disparities in health with a complex framework describing health outcomes [49]. Our study provides an explanation of the variables that are relevant to and usable by policy makers who can influence fundamental causes of health disparities with policy that addresses underlying causes of systemic racism including reallocation of scarce resources.

## Data Availability

Publicly available datasets were analyzed in this study. The data can be found here: https://www.countyhealthrankings.org/explore-health-rankings/georgia/data-and-resources; https://dph.georgia.gov/covid-vaccine; and https://experience.arcgis.com/experience/3d8eea39f5c1443db1743a4cb8948a9c.
